# Cytotoxic, Anti-Proliferative and Apoptosis Activity of l-Amino Acid Oxidase from Malaysian *Cryptelytrops purpureomaculatus* (CP-LAAO) Venom on Human Colon Cancer Cells

**DOI:** 10.3390/molecules23061388

**Published:** 2018-06-08

**Authors:** Syafiq Asnawi Zainal Abidin, Pathmanathan Rajadurai, Md. Ezharul Hoque Chowdhury, Iekhsan Othman, Rakesh Naidu

**Affiliations:** Jeffrey Cheah School of Medicine and Health Sciences, Monash University Malaysia, Jalan Lagoon Selatan, Bandar Sunway, 47500 Selangor Darul Ehsan, Malaysia; syafiqnawi@gmail.com (S.A.Z.A.); drpathma@gmail.com (P.R.); md.ezharul.hoque@monash.edu (M.E.H.C.); iekhsan.othman@monash.edu (I.O.)

**Keywords:** l-amino acid oxidase, *Cryptelytrops purpureomaculatus*, cytotoxicity, apoptosis, human colon cancer cells

## Abstract

The aim of this study is to investigate the potential anti-cancer activity of l-amino acid oxidase (CP-LAAO) purified from the venom of *Cryptelytrops purpureomaculatus* on SW480 and SW620 human colon cancer cells. Mass spectrometry guided purification was able to identify and purify CP-LAAO. Amino acid variations identified from the partial protein sequence of CP-LAAO may suggest novel variants of these proteins. The activity of the purified CP-LAAO was confirmed with o-phenyldiamine (OPD)-based spectrophotometric assay. CP-LAAO demonstrated time- and dose-dependent cytotoxic activity and the EC_50_ value was determined at 13 µg/mL for both SW480 and SW620 cells. Significant increase of caspase-3 activity, reduction of Bcl-2 levels, as well as morphological changes consistent with apoptosis were demonstrated by CP-LAAO. Overall, these data provide evidence on the potential anti-cancer activity of CP-LAAO from the venom of Malaysian *C. purpureomaculatus* for therapeutic intervention of human colon cancer.

## 1. Introduction

Cancer remains as one of the major causes of death and accounts for seven million deaths worldwide [1]. Colon cancer represents the second highest cause of cancer-related deaths in the United States with a death toll estimated around 50,830 in 2013 [2]. It was classified as second most frequent cancer in Malaysia, representing 12.3% of the population and trailing behind breast cancer which accounts for 18.1% of the population. The survival rate of colon cancer patients was lower in Malaysia compared to Singapore, United Kingdom, and United States [3]. Therefore, continued research into the treatment and management of the disease remains critical. Currently, various approaches have been used for cancer treatment, including colorectal cancer, for instance via surgery, chemotherapy, radiotherapy, and the much recent targeted therapy that involves immunotherapy and epigenetic therapy [2,4]. Standard regimens in treating colon cancer are the administration of cytotoxic chemotherapy drugs such as FOLFOX (folinic acid, fluoroacil, and oxaliplatin), FOLFIRI (folinic acid, fluoroacil, and irinotecan), and FOLFOXIRI (folinic acid, fluoroacil, oxaliplatin, and irinotecan) [5]. However, application of chemotherapeutic drugs has several known side effects including skin toxicity, nausea, vomiting, and hair loss. There are several novel therapeutic agents being developed for the treatment of colon cancer such as cancer stem cell inhibitors, BRAF and PI3K inhibitors, and the anti-RAS reovirus Reolysin^®^ [5]. Additionally, nature provides a rich source for new and alternative cancer therapeutic drug candidates due to the mega-biodiversity of natural chemical compounds or protein and polypeptides found in millions of flora and fauna species.

Malaysia’s rich natural resources may hold answers for novel and effective anti-cancer drug candidates. Snake venom is one of many abundant natural resources that can be obtained from locally found snakes in the country. The complexity and mixture of multiple compounds and proteins/polypeptides found in the snake venom, which contribute towards the venom action in killing and immobilizing its prey, can be an important source of therapeutic compounds or proteins. Snake venom l-amino acid oxidase (LAAO) have been studied for its anti-cancer potential through cytotoxic and apoptotic activity in various cancer cell lines including human promyelocytic leukemia cells (HL-60) [6,7,8], human T cell leukemia (jurkat) [8] human cervical adenocarcinoma (HeLa) [9,10], human lung adenocarcinoma (A549) [11], human breast adenocarcinoma (MCF-7) [12], human gastric and colon adenocarcinoma (MKN45 and RKO) [13], and Ehrlich ascites tumor cells [7]. The authors noted that the cytotoxic effects were mainly driven by the production of H_2_O_2_ by LAAO activity. Despite many studies on the anti-cancer potential of LAAO, none has been performed on LAAO from *C. purpureomaculatus* (CP-LAAO) and human colon cancer cells. Therefore, the present study is aimed to investigate the cytotoxic activity, anti-proliferative, and induction of apoptosis by CP-LAAO against primary and metastatic human colon cancer cells.

## 2. Results

### 2.1. Cytotoxic Screening of C. purpureomaculatus Crude Venom

**Figure 1:**
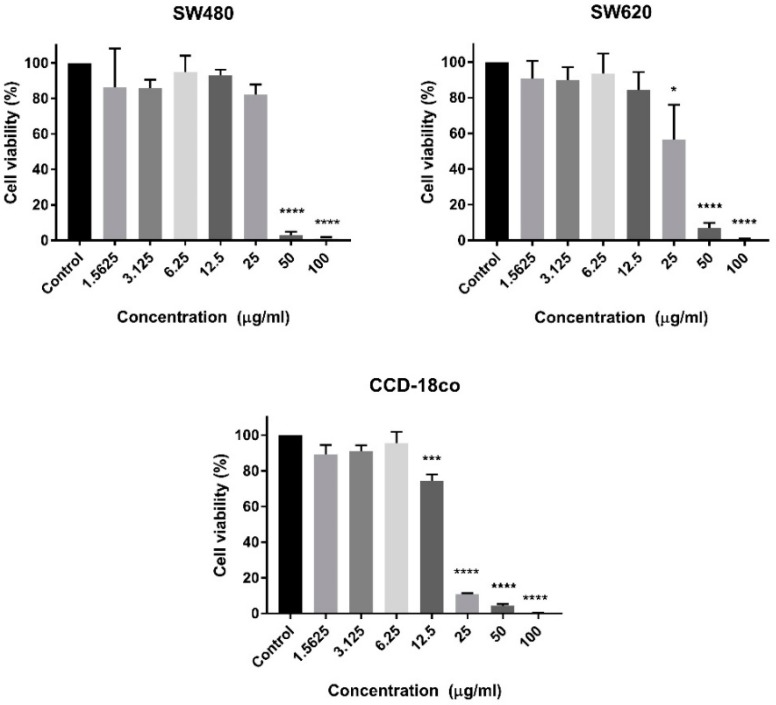
The cytotoxic effects of *C. purpureomaculatus* crude venom at different concentrations on SW480, SW620, and CCD-18co cell lines compared to untreated sample (control) after 72 h incubation. Data are presented as mean ± SD from three independent experiments. Percentage of cell viability and comparison between datasets were statistically analyzed using One Sample *t*-tests. Asterisks indicate statistically significant differences between the means of the values obtained with treated vs. untreated cells (* *p* < 0.05, *** *p* ≤ 0.001 **** *p* < 0.0001).

The crude venom of *C. purpureomaculatus* demonstrated cytotoxic activity in all cell lines with EC_50_ values of 29.43 μg/mL, 23.19 μg/mL, and 15.99 μg/mL in SW480, SW620, and CCD-18co, respectively (Figure 1, Table 1).

### 2.2. Purification of C. purpureomaculatus LAAO

CP-LAAO were purified from the crude venom following a bioassay guided and mass spectrometry approach. Subfraction 9 was selectively cytotoxic against the human colon cancer cells and therefore were chosen for further purification. LC-MS/MS analysis of the collected fraction identified CP-LAAO from subfraction 9 as pure since there were no other proteins detected from the fractions (Table S1). Sodium dodecyl sulfate-polyacrylamide gel electrophoresis (SDS-PAGE) of the collected subfraction demonstrated a single band confirming its purity (Figure S1). To the best of our knowledge, CP-LAAO from *C. purpureomaculatus* has not been previously characterized. CP-LAAO was determined to be homologous with LAAO from *Bothriechis, Calloselasma, Vipera,* and *Cerastes* genus (Table S1). Protein identification and automated de novo sequencing were able to determine the partial protein sequence of CP-LAAO by comparing against homologous sequences from other snake species determined from the database. *De novo* sequencing by LC-MS/MS of the isolated CP-LAAO showed that there were three amino acid substitutions at position 55 (isoleucine replaced by arginine), 286 (glutamate replaced by lysine), and 416 (glutamine replaced by proline), compared to homologous LAAO sequences (Table S2, Figure S2).

### 2.3. l-amino Acid Oxidase Assay

**Figure 2:**
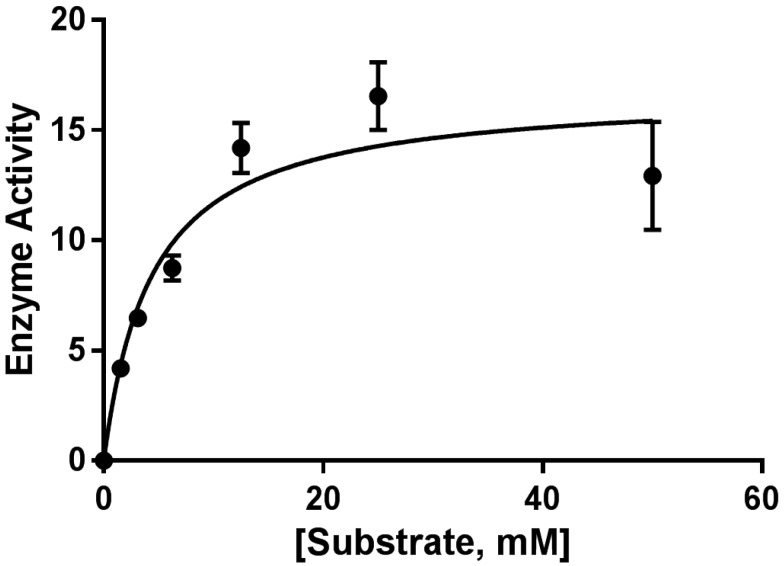
The effect of substrate concentration on enzyme activity. Increasing concentrations of l-leucine (substrate) were reacted with purified l-amino acid oxidase. The average velocity was then plotted against the substrate concentration using a non-linear regression fit Michaelis-Menten model to describe the data. The values for V_max_ and K_m_ were calculated using GraphPad Prism 7 software (version 7.0, Graphpad Software, La Jolla, CA, USA).

LAAO activity of *C. purpureomaculatus* crude venom and CP-LAAO was determined by *o*-phenyldiamine (OPD)-based microplate spectrophotometric assay. The Vmax value of the purified LAAO at every substrate (l-leucine) concentration was obtained from kinetic readings of the assay and was used to plot the Michaelis-Menten model of enzyme activity (velocity) against substrate concentration. V_max_ and K_m_ values of 16.77 mM/min and 4.376, respectively, were obtained from the model (Figure 2).

### 2.4. Cytotoxicity and Anti-Proliferative Activity of CP-LAAO

**Figure 3:**
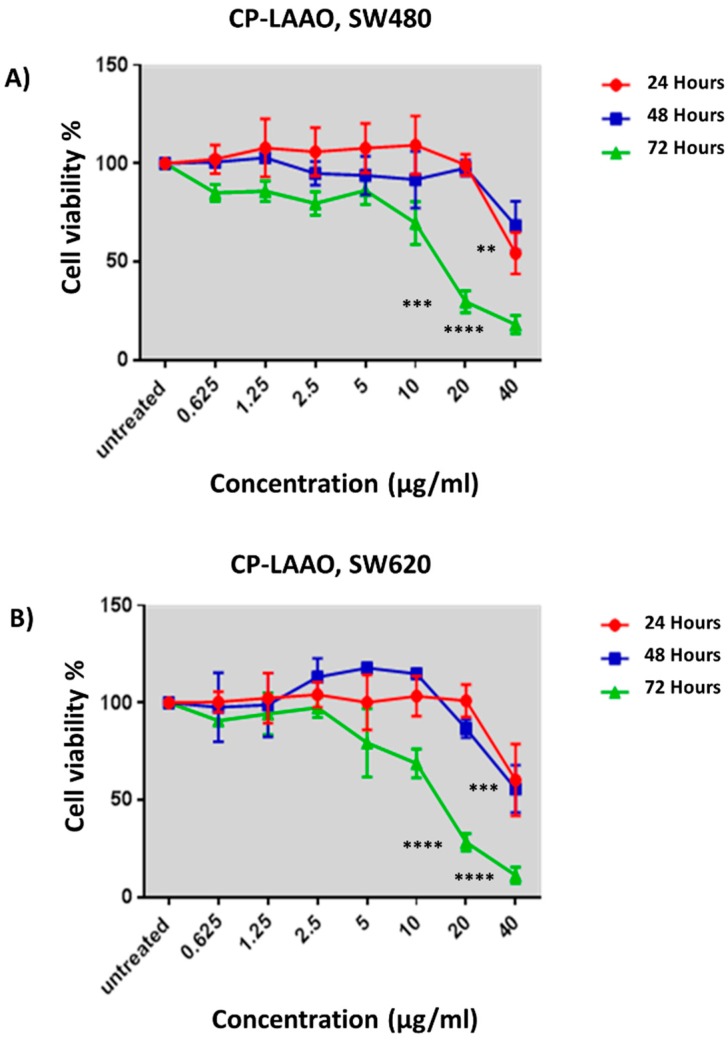
The anti-proliferative effects of purified CP-LAAO on SW480 (**A**) and SW620 (**B**), at 24, 48, and 72 h. The results are expressed as means of percentage cell viability over time. Experiments were performed in triplicates and results were compared between three independent experiments. ** *p* ≤ 0.01 and *** *p* ≤ 0.001, **** *p* ≤ 0.0001 indicates statistically significant differences between the means of values obtained with treated vs untreated cells.

Significant inhibition of cell proliferation was demonstrated when treated with 20 µg/mL and 40 µg/mL of CP-LAAO at 72 h in both SW480 (Figure 3A) and SW620 (Figure 3B) cells with cell viability of approximately 20–30%. Significant reduction of cell viability was noted at 24 h in SW480 cells treated with 20 µg/mL of CP-LAAO, but it was not significant at 48 h. In SW620 cells, treatment with 40 µg/mL CP-LAAO demonstrated significant reduction of cell viability at both 24 and 48 h. EC_50_ value of 13 µg/mL was obtained from the treatment of CP-LAAO in both cell lines at 72 h. The EC_50_ value of the normal colon cells was approximately 1.1 fold higher compared to SW480 and SW620. Overall, cytotoxic activity of CP-LAAO on the cancer cells were slightly higher in the cancer cells compared to the normal cells. However, the selective index (SX) values were noted at more than 100 (Table 2), which might still indicate a selective cytotoxic effect on the cancer cells.

### 2.5. Apoptotic Activity Induced by CP-LAAO on SW480 and SW620 Cells

**Figure 4:**
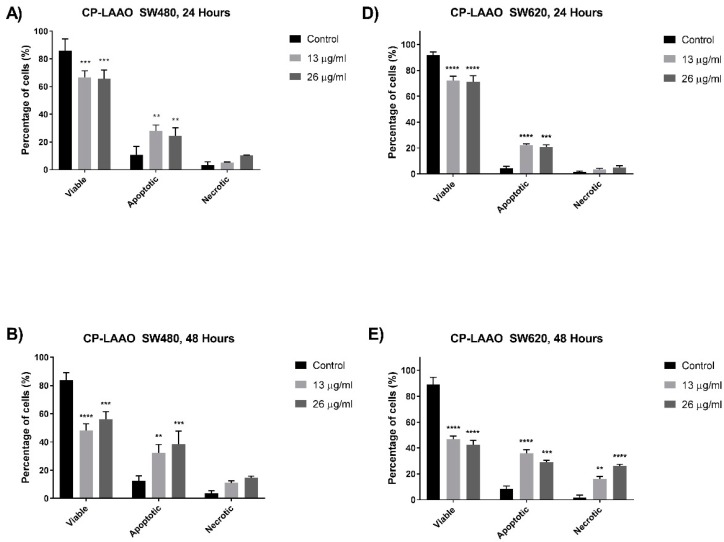

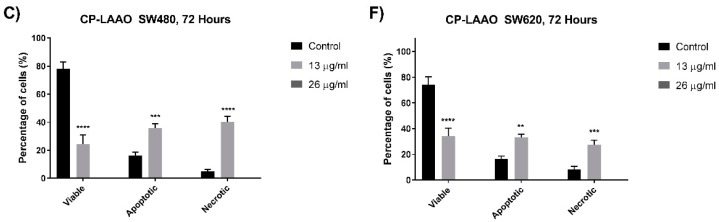
Percentage of viable, apoptotic, and necrotic cells in *C. purpureomaculatus* LAAO-treated SW480 (**A**–**C**) and SW620 (**D**–**F**) cells. Cells were treated with CP-LAAO for 24, 48, and 72 h. Treated and untreated cells (control) were double stained with annexin-V and propidium iodide and a minimum of 200 cells were counted per sample and the percentage of cells from each population (viable, apoptotic, and necrotic) was calculated. Experiments were performed in duplicates and comparable results were obtained from three independent experiments (*n* = 3). Comparison between data sets were performed using One Sample *t*-tests and asterisks indicate statistically significant (** *p* ≤ 0.01, *** *p* ≤ 0.001, **** *p* ≤ 0.0001) differences between data sets for each treatment dose. No data were obtained for SW480 and SW620 treatment with 26 µg/mL of CP-LAAO at 72 h.

Morphological changes associated with apoptosis on SW480 and SW620 cells were assessed using annexin V-FITC and propidium iodide staining. Cells were quantified and grouped into viable, apoptotic, and necrotic cells. CP-LAAO treatment on SW480 (Figure 4A–C) and SW620 (Figure 4D–F) cells resulted in a significant decline of viable cell population at 24 h by 10–20% at both doses of 13 µg/mL and 26 µg/mL of the treatment concentration. Percentage of viable cells further declined by 30–40% in both SW480 and SW620 at 48 h when treated with 13 µg/mL and 26 µg/mL of CP-LAAO. Significant increases of apoptotic cells were demonstrated following 24 h of CP-LAAO treatment, whereby, approximately 20–25% of cells had undergone apoptosis in both SW480 and SW620 cells at treatment concentrations of 13 µg/mL and 26 µg/mL. Forty-eight hours of post-treatment with 13 µg/mL of CP-LAAO demonstrated further increases of apoptotic cells in SW480 (approximately 30%) and SW620 (approximately 38%) cells. When the treatment dose was increased to 26 µg/mL at 48 h, the apoptotic cell population in SW480 and SW620 was approximately 38% and 30%, respectively. However, a significant increase of necrotic cells to approximately 18–24% of the total cell population was demonstrated following treatment with 13 µg/mL of CP-LAAO in SW480 and SW620 cells at 48 h. In SW620 cells, the necrotic cell population was significantly increased to approximately 34% of the total cell population when treated with 26 µg/mL of CP-LAAO at 48 h. Seventy-two hours of 13 µg/mL of CP-LAAO treatment in both cell lines caused further reductions of the viable cell population to approximately 20–30%, while the apoptotic cell population remained at approximately 30–35% of the total cell population. Necrotic cell population continued to increase significantly at 72 h of 13 µg/mL treatment to 35–40% of the total cell population in both cell lines. No data was obtained at 72 h when the dose was increased to 26 µg/mL. In summary, CP-LAAO appeared to induce apoptosis at 24 h after treatment for SW480 and SW620 cells in a time- and dose-dependent manner. The percentage of necrotic cells at 24 h was also relatively low compared with other time points.

### 2.6. CP-LAAO on Caspase-3 Activity of Treated SW480 and SW620 Cells

**Figure 5:**
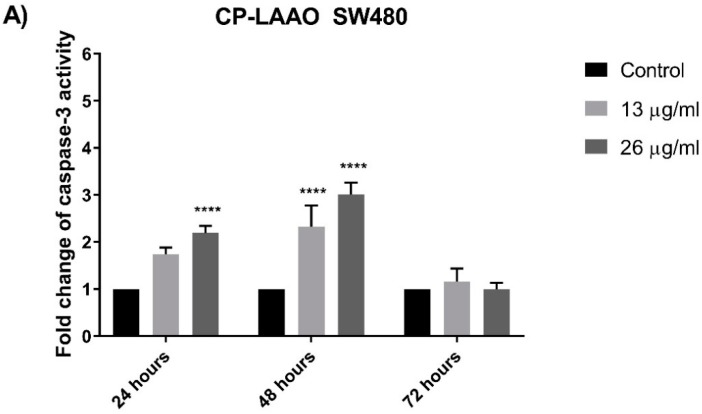

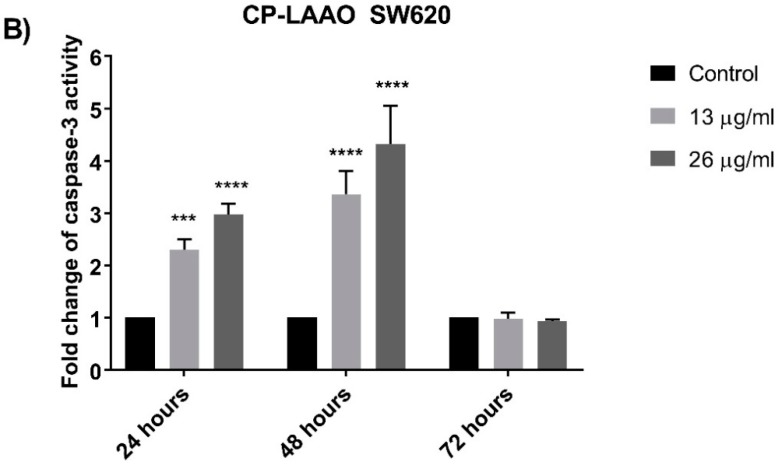
Caspase-3 activity in CP-LAAO-treated SW480 (**A**) and SW620 (**B**) cells measured at 24, 48, and 72 h. Experiments were performed in duplicates and results compared between three independent experiments (*n* = 3). Results were analyzed using One Sample *t*-tests between the ratios of means of caspase-3 activity of treated samples over untreated samples. Asterisks indicate statistically significant data (*** *p* ≤ 0.001, **** *p* ≤ 0.0001). Error bars represent standard deviation (SD).

Caspase-3 activity peaked at 48 h in both SW480 and SW620 cells when treated with 13 µg/mL and 26 µg/mL of CP-LAAO in a dose-dependent manner. The caspase-3 activity at 48 h was significantly higher compared to the caspase-3 activity at 24 h in SW480 (1.5–2 fold higher) and SW620 (2.5–3 fold higher) (Figure 5A,B). However, there was no significant increase of caspase-3 activity at 72 h in both cell lines when compared to untreated cells.

### 2.7. Quantification of Bcl-2 Protein Concentration on CP-LAAO Treated SW480 and SW620 Cells

**Figure 6:**
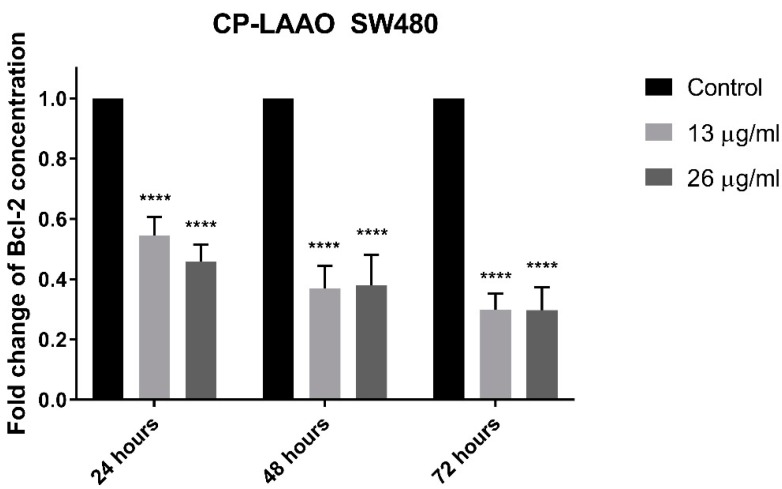

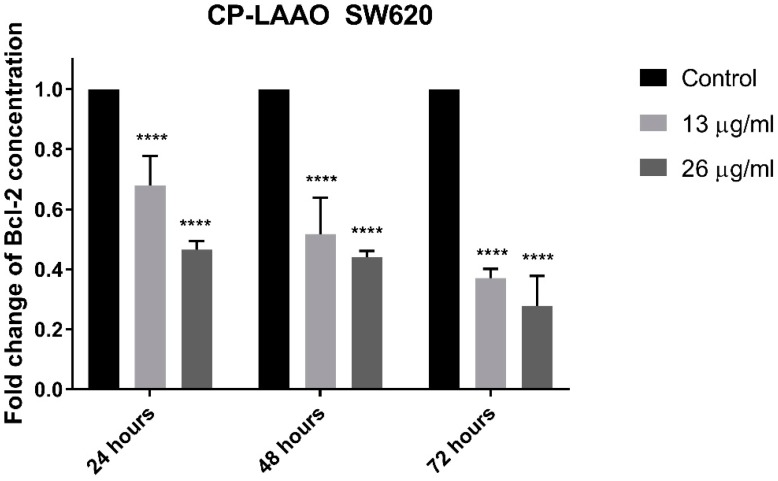
Bcl-2 protein concentration in CP-LAAO-treated SW480 (**A**) and SW620 (**B**) cells measured at 24, 48, and 72 h. Experiments were performed in duplicates and results compared between three independent experiments (*n* = 3). Results were analyzed using One Sample *t*-tests between the ratios of means of Bcl-2 protein concentration of treated samples over untreated samples. Asterisks indicate statistically significant data (**** *p* ≤ 0.0001). Error bars represent standard deviation (SD).

Treatment of SW480 and SW620 cells with 13 µg/mL and 26 µg/mL of CP-LAAO demonstrated a significant and progressive reduction of Bcl-2 concentration from 24 to 72 h of post-treatment (Figure 6A,B).

## 3. Discussion

In this current study, we have examined the cytotoxic activity of the Malaysian *C. purpureomaculatus* crude venom and CP-LAAO. Cytotoxic evaluation was conducted on primary (SW480) and metastatic (SW620) human colon cancer cells and normal human colon cells (CCD-18co). The crude venom of *C. purpureomaculatus* was found to effectively reduce the cell population viability of more than 50% at a concentration of more than 20 µg/mL. This finding was expected since crude venom is a mixture of different active proteins and therefore may interact and induce cytotoxic activity in all cells without being selectively cytotoxic. However, it is believed that this was the first study that has reported cytotoxic activities on human colon cancer cells due to the venom of Malaysian *C. purpureomaculatus.* CP-LAAO was purified from the crude venom and several studies have demonstrated anti-cancer activity of LAAO on several human cancer cell lines [9,14,15]. However, none have reported on human colon cancer cells.

Purification of CP-LAAO *C. purpureomaculatus* venom has not been reported previously. However, the presence of CP-LAAO in the crude venom was confirmed by our previous study [16]. Identification of the purified CP-LAAO on SDS-PAGE indicated a single protein chain (single subunit), as highlighted by the single band in both reduced and non-reduced conditions of both proteins. This identification was consistent with several reports the venom of other pit viper species, wherein, LAAO consist of single subunit proteins [17,18]. The molecular weight of CP-LAAO based on the SDS-PAGE was comparable to other known LAAO from the database which is approximately in the range of 55–60 kDa. From de novo sequencing of the isolated-CP-LAAO using LC-MS/MS and PEAKS software, we were able to construct the partial protein sequence of the protein. The peptide sequences were found to be homologous to proteins from other viper/pit viper species from the large *Serpentes* database in SwissProt/UniProt (2584 reviewed proteins, 64,430 unreviewed proteins) [19]. This was because LAAO from *C. purpureomaculatus* had never been identified and therefore the information was not available in the database.

The partial sequence of CP-LAAO was constructed by comparing the identified peptides from LC-MS/MS and de novo sequencing with the homologous protein from other viper/pit viper species. The partial sequences were able to highlight several amino acid variants in the peptide sequence. Based on the CP-LAAO partial sequence, there were three amino acid substitutions at positions 55, 286, and 416, where arginine replaced isoleucine, lysine replaced glutamate, and proline replaced glutamine, respectively. The significance of the substitutions at positions 55 and 416 is unknown; however, Lys-286 may be essential to the LAAO cofactor, flavo-adenine-nucleotide (FAD), binding domain. This is supported by sequential evidence provided in UniProt database of LAAO from other species such as *Calloselasma rhodostoma* [P81382 OXLA_CALRH], *Cerastes cerastes* [X2JCV5 OXLA_CERCE], and *Trimeresurus stejnegeri* [Q6WP39 OXLA_TRIST] that contain FAD binding sites at nearby positions [20]. FAD is a non-covalently bound cofactor of most snake venom LAAO and is essential in the production of hydrogen peroxide (H_2_O_2_) during the enzymatic reaction of LAAO [21,22]. It is possible that changes on FAD site could affect the cytotoxic activity of LAAO on cancer cells since the production of H_2_O_2_ from the enzyme was identified to contribute towards cytotoxicity and apoptotic activity in several cell lines, such as HL60 (human promyelocytic cell), HeLa (cervical cancer cells), MM6 (human monocytic cell), and human leukemia T-cells [7,8,9,23]. The purified CP-LAAO was demonstrated to be active based on the activity assay performed against substrate l-leucine in this study.

Anti-proliferative assay was performed over three time points to determine the potential of CP-LAAO in inhibiting SW480 and SW620 cell growth. CP-LAAO demonstrated significant cytotoxic activity in SW480 and SW620 cells at 72 h from 20 µg/mL onwards, compared with CCD-18co. EC_50_ value of 13 µg/mL was obtained from the treatment of CP-LAAO in both SW480 and SW620. It is believed that the present data represents the first evidence of CP-LAAO cytotoxic activity in human colon cancer cells. Several LAAOs from other snake species have been isolated and demonstrated cytotoxic activity to cancer cell lines including MCF-7 [12], MKN-45 [13], A549 [11], HL-60 [7,8], and HeLa [9]. The EC_50_ value of CP-LAAO obtained in this present study was higher compared with LAAOs isolated from *Bothrops moojeni*, which showed an EC_50_ value of 8 µg/ml in HL-60 cells [7]. However, CP-LAAO had higher cytotoxic activity than LAAOs isolated from *Bothrops atrox* and *Agkistrodon acutus*, which demonstrated EC_50_ values of 50 µg/mL in HL-60 cells and 20 µg/mL in HeLa cells, respectively [8,9]. Additionally, the cytotoxicity of CP-LAAO was influenced by the dose and treatment length.

Apoptosis pathways are often dysregulated in the progression of cancerous cells [24]. Therefore, targeting the apoptosis mechanism is deemed important in the development of effective anti-cancer therapeutic agents. In this present study, several assays were utilized to determine the induction of apoptosis via morphological and biochemical assessments. Quantitative analysis of the cells was performed based on the morphological features to investigate the apoptotic cell population. A significant increase in the apoptotic cell population was observed from SW480 and SW620 cells treated with CP-LAAO at 24 h and peaked at 48 h. However, CP-LAAO treatment caused a significant increase of necrosis, especially in SW620, where it reached approximately 20% and 30% of the cell population at 48 h of 13 µg/mL and 26µg/mL of treatment concentration, respectively. Taken together, the quantitative morphological assessment of treated SW480 and SW620 cells indicate that CP-LAAO had the capability of inducing apoptosis in a time- and dose- dependent manner.

The induction of apoptosis and the downstream signaling is mediated by several important proteins such as caspase-3 and Bcl-2 [25]. The results clearly demonstrated that caspase-3 activity was significantly increased in both colon cancer cell lines (SW480 and SW620) over the basal level of untreated (control) cells after exposure with CP-LAAO at 24 h and peaked at 48 h. Strikingly, there was no significant increase of caspase-3 activity after 72 h from all treatment in both cell lines when compared with the untreated cells. These results demonstrated a caspase-3-dependent apoptosis in SW480 and SW620 cells. Therefore, it could be suggested that CP-LAAO was able to induce apoptosis in both cell lines. The ability of LAAO to induce apoptosis in human cancer cell lines was evident in HeLa [15,26], MM6 [12], Jurkat cells [27], and RBR 17T (human malignant glioma cells) [28]. Furthermore, activation of caspase-3 were often demonstrated in cancer cell lines upon LAAO exposure [8,26,29]. Besides caspase-3, Bcl-2 is a key protein in apoptosis induction. Bcl-2 regulates mitochondrial membrane permeability and integrity, in addition to suppression of cytochrome c release [30,31]. It was demonstrated that overexpression of Bcl-2 may protect the cancer cell from cell death while decrease in expression may trigger apoptosis [32]. In this present study, treatment of CP-LAAO on SW480 and SW620 cells resulted in a significant reduction of Bcl-2 cellular protein concentration in a dose- and time-dependent manner. The present findings are supported by several studies that demonstrated reduced expression of Bcl-2 proteins in HepG2 (human hepatocyte carcinoma) and HL-60 (human promyelocytic leukemia) cells when treated with LAAO [33]. Therefore, the present findings suggest that CP-LAAO induces apoptosis by decreasing Bcl-2 expression and increased caspase-3 activity.

## 4. Materials and Methods

### 4.1. Snake Venom

Crude *C. purpureomaculatus* venom were obtained from Mr. Zainuddin Ismail, a private and licensed snake handler from Perlis, Malaysia. The venom was carefully collected in a sterile container covered with parafilm and kept on ice during transportation. Prior to usage, the venom was frozen at −80 °C followed by lyophilization process. Dried venoms were weighed, labelled, and stored at −20 °C prior to use.

### 4.2. Protein Quantification by Bichinchoninic Acid Assay (BCA Assay)

Protein quantification was performed with Pierce BCA Protein Assay Kit (Thermo Fisher Scientific, Waltham, MA, USA) and as instructed by the manufacturer’s protocol. Serially diluted bovine serum albumin (BSA) was used as a standard (60–2000 μg/mL). The absorbance was measured at 562 nm with a microplate spectrophotometer reader (BioTek™ EON™ Microplate Spectrophotometers, Fisher Scientific, USA).

### 4.3. Preparation of Crude Venom and Purified CP-LAAO for Cytotoxic and Apoptosis Evaluation

Crude venom and purified CP-LAAO were quantified using a Pierce© BCA assay kit. The crude venom was serially diluted in the appropriate media at a concentration range of 1.56 μg/mL, 3.125 μg/mL, 6.25 μg/mL, 12.5 μg/mL, 25 μg/mL, 50 μg/mL, and 100 μg/mL (two-fold dilution) prior to cell treatment. CP-LAAO was subjected to caspase-3 and Bcl-2 assays and was prepared in the appropriate cell culture media at the following concentrations; CP-LAAO; 13 μg/mL (1× EC_50_) and 26 μg/mL (2× EC_50_) in SW480 and SW620. Similar concentrations were used for CP-LAAO for morphological assessment using Annexin-V and propidium iodide.

### 4.4. Cell Culture and Maintenance

Primary (SW480, ATCC^®^ CCL-228 ™) and metastatic (SW620, ATCC^®^ CCL-227 ™) colon cancer cell lines and normal colon cell line (CCD-18co, ATCC^®^ CRL-1459 ™) were obtained from the American Type Culture Collection (ATCC, Manassas, VA, USA). SW480 and SW620 cells were maintained in Leibovitz L-15 medium (Corning Life Sciences, Corning, NY, USA) supplemented with 10% fetal bovine serum and 1% antibiotics; penicillin and streptomycin, in a 37 °C incubator without CO_2_. CCD-18co cells were maintained in Eagles Minimum Essential Medium (EMEM) (Corning Life Sciences, USA) supplemented with 10% fetal bovine serum and 1% antibiotics, in a 37 °C incubator with CO_2_. Cells were frequently monitored to ensure a normal and consistent morphology. While maintaining proper aseptic techniques, the cells were subcultured every 2–3 days or upon 90% confluency and the culture medium replenished within a biosafety cabinet (Labculture © Class II, Type A2 Biological Safety, Esco Technologies Inc., Horsham, PA, USA).

### 4.5. Cytotoxic and Anti-Proliferative Assay

The cells were seeded in a flat-bottomed microtitre 96-well plates (Nunc, Roskilde, Denmark) at a concentration of 70,000 cells/mL together with appropriate culture media in triplicates and incubated for 24 h at 37 °C in a humidified incubator to allow the cells to adhere to the bottom of the wells. After 24 h, the media was aspirated and replaced with fresh media containing different treatment doses of the crude venom or purified CP-LAAO. Control cells were treated with only double distilled water. The cells were incubated for 72 h at 37 °C, with and without 5% CO_2_ for CCD-18co and SW480/620, respectively. To assess the cell viability, colorimetric 3-(4, 5-dimethylthiazol-2-yl)-2, 5-diphenyltetrazolium bromide (MTT) assay was performed. Briefly, the medium was aspirated and 100 μL of medium with MTT solution (0.5 mg/mL in PBS) was added to every well and incubated for 4 h at 37 °C. The MTT was aspirated and 100 μL dimethyl sulfoxide (DMSO, Molecular Biology Grade, Sigma-Aldrich, St. Louis, MO, USA) was added into each well to dissolve the formazan crystals. The absorbance of the blue formazan was read at 570/650 nm wavelengths using a microplate spectrophotometer (BioTek™ EON™ Microplate Spectrophotometers, Fisher Scientific, USA). The percentage of cell viability was calculated as follows:

$$Cell\;viability\;\%=\;\frac{Average\;absorbance\;of\;treated\;cells}{average\;absorbance\;of\;untreated\;cells\;\left(control\right)}\;\times 100$$

EC_50_ values were generated from GraphPad Prism 7 software (Graphpad Software, La Jolla, CA, USA) with non-linear regression curve fits of the data, where EC_50_ value was the venom concentration required to reduce cell viability by at least 50% of the cell population. The overall EC_50_ from the cytotoxic assay was calculated based on EC_50_ values from 3 independent experiments at 72 h. The cytotoxic assay was performed on the crude, fractions, subfractions and purified proteins of the venom. The anti-proliferative activity was performed using MTT assay at three time points; 24, 48, and 72 h to determine the time- and dose-dependent effect.

### 4.6. Selectivity Index

The degree of selectivity of the purified proteins towards cancer cells was determined by its selectivity index value, which was adapted from studies by Popiolkiewicz et al., (2005) [34] and Chew et al., (2012) [35] and calculated from EC_50_ values obtained from the treatment of the purified proteins on colon cancer cell lines and normal colon cells. The index value was calculated using the following formula:

$$selectivity\;index\;\left(SX\right)\;=\;\frac{EC_{50}\;normal\;colon\;cell\;line}{EC_{50}\;colon\;cancer\;cell\;line}\times 100$$

Selectivity index value above 100 indicates a high cytotoxic selectivity of the purified proteins towards cancer cells compared to normal cells.

### 4.7. Purification of LAAO from C. purpureomaculatus Crude Venom

Fifty mg of crude venom was dissolved in Milli-Q water and later being centrifuged at 1,000 rpm for 5 min. Supernatants were collected and loaded into a C 26/100 column (GE Healthcare Life Sciences, Marlborough, MA, USA) that was manually packed with Sephadex G-50 Fine (GE Healthcare Life Sciences, USA) gel filtration medium, and mounted on a Äkta Prime Plus system. The column was equilibrated with 0.05 M ammonium acetate pH 6.8, which ran at a flow rate of 0.2 mL/min and elution of the peaks were monitored at 208 nm and automatically collected by the system. The collected fractions were pooled for another 3–4 runs and later freeze dried and re-dissolved in Milli-Q water prior use. Freeze-dried fraction 1 of *C. purpureomaculatus* venom was dissolved in ddH2O and centrifuged at 10,000 RPM before being loaded into a Jupiter© C-18 semi-preparative reverse-phase column (Phenomenex, Torrance, CA, USA, 10 μ, 300Å, 250 × 10 mm) mounted on an Agilent HPLC 1260 Infinity system (Agilent Technologies, Santa Clara, CA, USA). The column was equilibrated with 5% of 90% HPLC-grade acetonitrile with 0.1% formic acid at a flow rate of 1 mL/min and maximum pressure of 200 BAR. All fractions were automatically collected using the fraction collector. The fractions were eluted from the column with an increasing percentage of 90% acetonitrile in 0.1% formic acid using the following multiple step gradient: 5%, 0–5 min; 20%, 6–16 min; 25%, 17–27 min; 30%, 28–38 min, 35%, 39–49 min; 40%, 50–60 min; 45%, 61–71 min; 50%, 72–82 min; 100%, 83–92 min; and 5%, 93–113 min. LAAO were purified from subfraction 9 at retention time of approximately 54.5 min.

### 4.8. l-amino Acid Oxidase Activity Assay

The activity of CP-LAAO was performed through a spectrophotometric microplate assay as described by Kishimito and Takahashi [36] with modifications. Briefly, the assay was conducted in triplicates of 10 µL/well of enzyme solution and 90 µL/well of substrate solution in a 96-well plate. Reaction mixture contained l-amino acid (l-leucine) that was serially diluted at a range of 1.56 mM to 100 mM, 2 mM o-Phenyldiamine (OPD), 1 mg/mL of horseradish peroxidase (HRP) (Sigma-Aldrich, St. Louis, MO, USA), and LAAO in a total volume of 100 µL/well of 0.1 M Tris-HCl buffer (pH 7.8). The plate was incubated at 37 °C for 60 min and the reaction was terminated with 50 µL of 2 M HCl. The absorbance was measured by BioTek™ EON™ (Fisher Scientific, USA) microplate reader at 492/630 nm. In the kinetic experiment, absorbance was measured at 420/630 nm at time intervals of 5 min within 1 h at 37 °C. V_max_ value was obtained from Gen5 Data Analysis Software (version 2.0, BioTek, Winooski, VT, USA) bundled with the spectrophotometer. Michaelis-menten data were generated from GraphPad Prism 7 software (Graphpad Software, La Jolla, CA, USA) from enzyme activity (velocity) against substrate concentration plot.

### 4.9. In-Solution Tryptic Digestion

Approximately 0.5 mg of total protein was added into 1.5 mL tube and mixed with 25 μL of 100 mM ammonium bicarbonate (ABC), 25 μL of trifluroethanol and 1 μL of 200 mM dithiothreitol (DTT). The mixture was then briefly vortexed, and incubated at 60 °C for 1 h. Next, the protein in the tube was alkylated by adding 4.0 μL of 200 mM iodoacetamide (IAA), briefly vortexed, and incubated at room temperature in the dark (covered with foil) for 1 h. Subsequently, 1 μL of 200 mM DTT was added to the tube and incubated at room temperature in the dark for another 1 h. Milli-Q water and 100 mM ABC were then added to the protein to dilute the protein denaturant and raising the pH to pH 7–9, respectively. Trypsin solution was then added to the tubes on a ratio of 1:50, briefly vortexed and incubated overnight at 37 °C. The following day, 1 μL of formic acid was added to stop the trypsin activity, briefly vortexed, and left in a vacuum concentrator overnight to concentrate the digested proteins. Samples were kept at 20 °C prior to LC-MS/MS analysis.

### 4.10. Protein Analysis via Nanoflow Liquid Chromatography Coupled with Tandem Mass Spectrometry (Nanoflow-ESI-LC-MS/MS)

Digested peptides from in-solution tryptic digestion were loaded into an Agilent C18 300 Å Large Capacity Chip (Agilent Technologies, Santa Clara, CA, USA) and equilibrated with 0.1% formic acid in water (solution A). The peptides were then eluted from the column with an increasing gradient of 90% acetonitrile in 0.1% formic acid in water (solution B) by the following gradient; 3–50% solution B from 0–30 min, 50–95% solution B from 30–32 min, 95% solution B from 32–39 min and 95–3% solution B from 39–47 min. Q-TOF polarity was set at positive with capillary and fragmenter voltage being set at 2050 V and 300 V, respectively, and 5 L/min of gas flow with a temperature of 300 °C. The intact protein spectrum was analyzed in auto MS mode ranging from 110–3000 *m*/*z* for MS scan and 50–3000 *m*/*z* for MS/MS scan. The spectrum was then analyzed with Agilent MassHunter data acquisition software (version B.07.00, Agilent Technologies, Santa Clara, CA, USA) and then PEAKS 7.0 software (Bioinformatics Solutions Inc., Waterloo, ON, Canada).

### 4.11. Venom Protein and Peptide Identification by Automated de novo Sequencing (PEAKS Studio) and Data Analysis

Protein identification by automated de novo sequencing was performed using PEAKS Studio 7.0 (Bioinformatics Solution, Waterloo, ON, Canada). The SwissProt Serpentes database was used for protein identification and homology search by comparing the de novo sequence tag. Carbamidomethylation was set as fixed modification with maximum mixed cleavages at three. Parent mass and fragment mass error tolerance were set at 0.1 Da with monoisotopic as the precursor mass search type. Trypsin was selected as the digestion enzyme. False discovery rate (FDR) of 1% and protein score of −10 lgP > 20 were used for filtering out inaccurate proteins. PEAKS indicated that a −10 lgP score of greater than 20 was of relatively high in confidence as it targeted very few decoy matches above that threshold.

### 4.12. Sodium Dodecyl Sulfate-Polyacrylamide Gel Electrophoresis (SDS-PAGE)

SDS-PAGE was performed on a 12% polyacrylamide gel via method that has been previously described (Manns, 2005). SDS-PAGE was performed on a Hoefer SE260 vertical electrophoresis system (Hoefer, Inc. Holliston, MA, USA) until the loading dye front reached the bottom of gel. The gels were then stained with Blue Bandit Protein Stain™ (VWR Life Science AMRESCO, Radnor, PA USA) for 1 h and de-stained with distilled water. The gel was later scanned using a ChemiDoc XRS Imaging System (BioRad, Hercules, CA, USA).

### 4.13. Quantitative Analysis of Apoptotic Cells

Apoptosis detection was performed using the Annexin-V Apoptosis Detection Kit (Raybiotech Inc., Norcross, GA, USA) following the manufacturer’s instruction with slight modifications. SW480 and SW620 cells were plated and exposed to treatment for 24, 48, and 72 h. Twenty microlitre of the cell suspension were placed in the bottom of a clean 6-well plate and covered with a glass coverslip. Fluorescent detection was observed under an inverted fluorescence microscope (Olympus IX81 Inverted Fluorescence Microscope, Olympus, Shinjuku, Tokyo, Japan) using a dual filter set for FITC (green) and rhodamine (red). A minimum of 200 total targeted cells were counted per sample and the percentage of cells from each population (viable, apoptotic, and necrotic cells) was calculated according to the equation:

$$\%\;of\;cells=\;\frac{number\;of\;viable\;or\;apoptotic\;or\;necrotic\;cells}{200\;cells}\times 100$$

The morphological features of the stained cells were distinguished by the bound annexin V-FITC and PI. Apoptotic cells have bound annexin V-FITC and exhibited bright green staining in the plasma membrane and/or bright green and red staining (PI) in the plasma membrane and nuclei, respectively. Necrotic cells showed uniformly red in appearance.

### 4.14. Quantification of Caspase-3 Activity

Caspase-3 activity was analyzed and performed using the Caspase-3 Colorimetric Assay Kit (Raybiotech Inc., USA), following the manufacturer’s instruction. SW480 and SW620 were plated and exposed to treatment for 24, 48, and 72 h. The intensity of the color was measured at 400 nm by using a microplate spectrophotometer (BioTek™ EON™ Microplate Spectrophotometers, Fisher Scientific, USA). The data was presented in fold-change of absorbance from treated cells against absorbance from untreated cells:

$$\begin{array}{cc} Fold-change & =\left(Abs.\;reading\;\left(400nm\right)\;of\;treated\;cells\right)\\ & \;⁄\left(Abs.\;reading\;\left(400nm\right)\;of\;untreated\;cells\right) \end{array}$$

### 4.15. Quantification of Bcl-2 Cellular Protein Concentration Using Enzyme-Linked Immunosorbent Assay (ELISA)

Bcl-2 cellular protein concentration was quantified using the Human Bcl-2 Platinum ELISA kit (Affymetrix eBioscience, Vienna, Austria) following the manufacturer’s instruction. Briefly, SW480 and SW620 cells were cultured and treated for 24, 48, and 72 h. The color intensity was measured at 450 nm by using a microplate spectrophotometer (BioTek™ EON™ Microplate Spectrophotometers, Fisher Scientific, USA). Bcl-2 concentrations from the samples were obtained by comparing the absorbance obtained against the standards. Data was presented in fold-change of absorbance from treated cells against absorbance from untreated cells:

$$\begin{array}{cc} Fold-change & =\left(Abs.\;reading\;\left(400nm\right)\;of\;treated\;cells\right)\\ & \;⁄\left(Abs.\;reading\;\left(400nm\right)\;of\;untreated\;cells\right) \end{array}$$

## 5. Conclusions

In conclusion, CP-LAAO was purified from the crude venom of *C. purpureomacuatus* and demonstrated significant cytotoxic activity on SW480 and SW620 cells. Time-dependent analysis revealed that CP-LAAO demonstrated significant anti-proliferative effects at 48 and 72 h. SW480 and SW620 cells treated with CP-LAAO demonstrated significant induction of caspase-3 activity, significant reduction of Bcl-2 levels and classic morphological features that were consistent with apoptosis. Our findings suggest that CP-LAAO has potential anti-cancer activity and may be further investigated as a potential therapeutic agent for the treatment of colon cancer.

## Figures and Tables

**Table 1:** Average EC_50_ from SW480, SW620, and CCD-18co treated with *C. purpureomaculatus* crude venom for 72 h.

Cell Lines	EC_50_ (µg/mL) ± SD
SW480	29.43 ± 0.48
SW620	23.19 ± 1.57
CCD-18co	15.99 ± 1.20

**Table 2:** EC_50_ and selective index (SX) values of SW480, SW620, and CCD-18co treated with CP-LAAO at 72 h.

Cell Lines	EC_50_ Values (µg/mL) ± SD	Selective Index Value (SX)
**SW480**	13.56 ± 0.58	110.47
**SW620**	13.17 ± 0.77	113.74
**CCD-18co**	14.98 ± 2.67	-

## Supplementary Materials

The following are available online. Figure S1: SDS-PAGE of purified L-amino acid oxidase (reduced (R) and non-reduced (NR)) aligned with Pierce broad Range Spectra Multicolour protein ladder, Table S1: LC-MS/MS results of purified CP-LAAO, Table S2: Partial peptide sequences of CP-LAAO identified from LC-MS/MS and *de novo* sequencing, Figure S2: Partial amino acid sequence of *C. purpureomaculatus* LAAO identified from LCM-MS/MS.
