# Supplementary material for: Cytotoxic, Anti-Proliferative and Apoptosis Activity of l-Amino Acid Oxidase from Malaysian Cryptelytrops purpureomaculatus (CP-LAAO) Venom on Human Colon Cancer Cells
Source: Molecules. 2018 Jun 8;23(6):1388. doi: 10.3390/molecules23061388 (PMC6100610; doi:10.3390/molecules23061388)
Supplement: Supplementary file 1 [file molecules-23-01388-s001.pdf]

Supplementary File

Table S1. LC-MS/MS results of purified CP-LAAO.

Accession	-10lgP	Coverage (%)	#Peptides	#Unique	Avg. Mass	Description
A0A024BTN9I OXLA_BOTSC	252.91	24	11	2	56376	L-amino acid oxidase Bs29 (Fragment) Bothriechis schlegelii
P81382I OXLA_CALRH	221.31	35	11	4	58221	L-amino-acid oxidase Calloselasma rhodostoma
P0DI84I OXLA_VIPAA	215.1	21	10	3	54748	L-amino-acid oxidase Vipera ammodytes ammodytes
Q6WP39I OXLA_TRIST	200.14	25	10	2	58601	L-amino-acid oxidase Trimeresurus stejnegeri
X2JCV5I OXLA_CERCE	141.97	16	9	2	58557	L-amino acid oxidase Cerastes cerastes

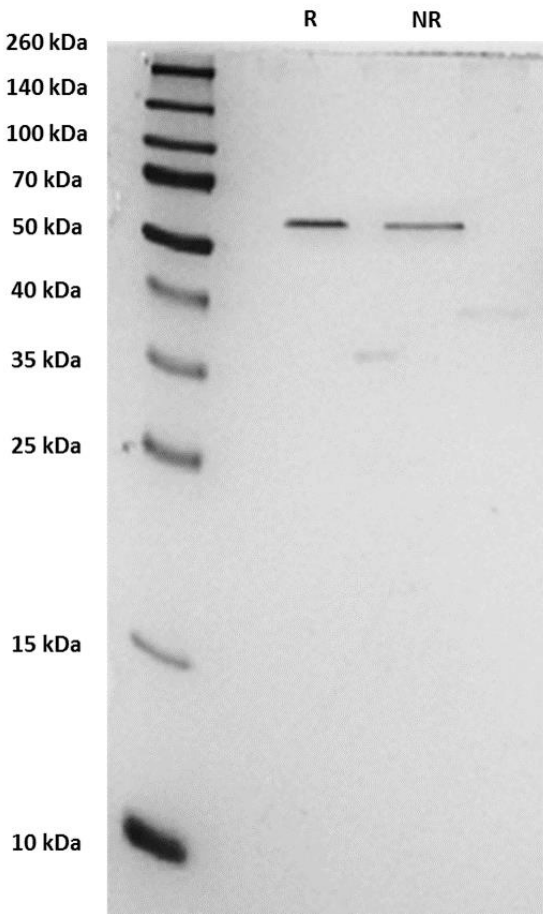

Figure S1. SDS-PAGE of purified L-amino acid oxidase (reduced (R) and non-reduced (NR)) aligned with Pierce Broad Range Spectra Multicolour protein ladder (E)

Table S2 Partial peptide sequences of CP-LAAO identified from LC-MS/MS and *de novo* sequencing

Protein Accession	Peptide	Unique	-10lgP	Mass	Length	ppm	m/z	z	RT
P81382|OXLA_CALRH	MNVFFMFSLLFLAALGSC(+57.02)ADDR.N	N	27.62	2524.195	22	-42	632.0296	4	4.62
Q6WP39|OXLA_TRIST	MNVFFMFSLLFLAALGSC(+57.02)ADDR.N	N	27.62	2524.195	22	-42	632.0296	4	4.62
Q6WP39|OXLA_TRIST	A.DDRNPLEEC(+57.02)FR.E	N	64.97	1449.631	11	1.9	484.2184	3	8.02
Q6WP39|OXLA_TRIST	A.DDRNPLEEC(+57.02)FRETDYEEFLEIAR.N	N	83.83	2945.314	23	-0.5	737.3353	4	12.89
P0DI84|OXLA_VIPAA	K.TS(+79.97)NPKHVVVVGAGMS(+79.97).G	Y	19.82	1641.699	15	46.6	411.4511	4	5.11
A0A024BTN9|OXLA_BOTSC	K.HVVIVGAGMSGLSAAYVLAGAGHQVTVLEASER.A	Y	125.43	3248.698	33	-5.3	813.1774	4	13.32
A0A024BTN9|OXLA_BOTSC	G.LSAAYVLAGAGHQVTVLEASER.A	N	51.43	2241.175	22	-1.8	748.0644	3	11.36
A0A024BTN9|OXLA_BOTSC	K.HVVR(**sub I**)VGAGMSGLSAAYVLAGAGHQVTVLEASER.A	Y	34.74	3291.715	33	-9.1	823.9285	4	12.65
P81382|OXLA_CALRH	K.HVVIVGAGMAGLSAAYVLAGAGHQVTVLEASERPGGR.V	Y	38.6	3599.899	37	-3.2	720.9849	5	13.49
A0A024BTN9|OXLA_BOTSC	K.EGWY(-18.01)ANLGPMR(+14.02).L	N	16.65	1288.602	11	33.1	645.3298	2	12.18
P0DI84|OXLA_VIPAA	K.EGWY(-18.01)ANLGPMR(+14.02).I	N	16.65	1288.602	11	33.1	645.3298	2	12.18
P0DI84|OXLA_VIPAA	R.KFGLNLNEFSQENDNAWYFIK.N	Y	16.84	2576.233	21	-2.7	645.0639	4	24.93
P81382|OXLA_CALRH	K.DPGLLKYPVKPSEAGKSAGQLYEESLGK.V	Y	18.84	2960.549	28	58.3	741.1877	4	21.45
Q6WP39|OXLA_TRIST	R.FDEIVDGMDK(+27.99)LPTSMYR.A	Y	16.01	2043.928	17	5	682.3201	3	8.6
A0A024BTN9|OXLA_BOTSC	K.LPTSMYQAI.E	N	16.34	1022.511	9	6.1	512.2657	2	9.61
X2JCV5|OXLA_CERCE	K.LPTSMYQAI.Q	N	16.34	1022.511	9	6.1	512.2657	2	9.61
X2JCV5|OXLA_CERCE	E.VTVTYQTPAK.N	Y	50.75	1106.597	10	-0.7	554.3055	2	5.87
X2JCV5|OXLA_CERCE	K.K(**sub E**)VTVTYQTPAK.N	Y	22.86	1234.692	11	-3.1	618.3514	2	5.42
A0A024BTN9|OXLA_BOTSC	K.IFLTC(+57.02)TK.K	N	53.04	881.4681	7	0.5	441.7415	2	7.18
Q6WP39|OXLA_TRIST	K.IFLTC(+57.02)TK.K	N	53.04	881.4681	7	0.5	441.7415	2	7.18
X2JCV5|OXLA_CERCE	K.IFLTC(+57.02)TK.K	N	53.04	881.4681	7	0.5	441.7415	2	7.18
A0A024BTN9|OXLA_BOTSC	K.FWEDDGIHGGK.S	N	64.75	1259.557	11	1.5	420.8603	3	6.71
P81382|OXLA_CALRH	K.FWEDDGIHGGK.S	N	64.75	1259.557	11	1.5	420.8603	3	6.71
P0DI84|OXLA_VIPAA	K.FWEDDGIHGGK.S	N	64.75	1259.557	11	1.5	420.8603	3	6.71
X2JCV5|OXLA_CERCE	K.FWEDDGIHGGK.S	N	64.75	1259.557	11	1.5	420.8603	3	6.71
Q6WP39|OXLA_TRIST	R.EDEGIHGGKSTTDLPSR.F	N	17.69	1797.849	17	-0.3	450.4695	4	4.36

P81382|OXLA_CALRH	G.VIIAYGIGDDANFFQALDFKDC(+57.02)ADIVFNDLSLIHQLPK.K	Y	34.59	4295.161	38	-8.9	1074.788	4	17.15
P0DI84|OXLA_VIPAA	K.EEIP(**sub Q**)SFC(+57.02)YPSMIQK.W	Y	34.52	1727.79	14	25.3	576.9518	3	8.9
A0A024BTN9|OXLA_BOTSC	G.ITTFTPYQFQHFSEA.L	N	23.41	1815.847	15	-3.1	908.9279	2	10.85
Q6WP39|OXLA_TRIST	G.ITTFTPYQFQHFSEA.L	N	23.41	1815.847	15	-3.1	908.9279	2	10.85
X2JCV5|OXLA_CERCE	G.ITTFTPYQFQHFSEA.L	N	23.41	1815.847	15	-3.1	908.9279	2	10.85
P81382|OXLA_CALRH	R.IYFAGEYTAQAHGWIDSTIK.S	N	19.99	2270.101	20	-5.9	757.7031	3	27.83
X2JCV5|OXLA_CERCE	R.IYFAGEYTAQAHGWIDSTIK.S	N	19.99	2270.101	20	-5.9	757.7031	3	27.83

1 MNVFFMFSLL FLAALGSCAD DRNPLEECFR ETDYEEFLEI AR**xxxx**TSNP KHVV**R**VGAGM SGLSAAYVLA GAGHQVTVLE

81 ASERPGGR**xx xxxxxx**EGWY ANLGPMR**xxx xxxxxxxxxx** KFGLNLNEFS QENDNAWYFI K**xxxxxxxxx x**DPGLLKYPV

161 KPSEAGKSAG QLYEESLGK**x xxxxxxxxxx xxxxxxxxxx xxxxxxxxxx xxxxxxxxxx xxxxxxxxxx xxxxxxxxxx**

241 **xxxxxxxx**FD EIVDGMDKLP TSMYQAI**xxx xxxxxxxxxx xxxxx****K**VTV TYQTPAK**xxx xxxxxxxxxx xxxxxxxxxx**

321 **xxxxxxxxxx xxxxxxxxxx xxx**IFLTCTK KFWEDDGIHG GKSTTDLPSR **xxxxxxxxxx xxxx**VIIAYG IGDDANFFQA

401 LDFKDCADIV FNDLSLIHQL PKEEI**P**SFCY PSMIQK**xxxx xxxxxx**ITTF FTPYQFQHFS EA**xxxxxxx**I YFAGEYTAQA

481 HGWIDSTIK**x xxxxxxxxxx xxxxxxxxxx xxxxxxxxxx**

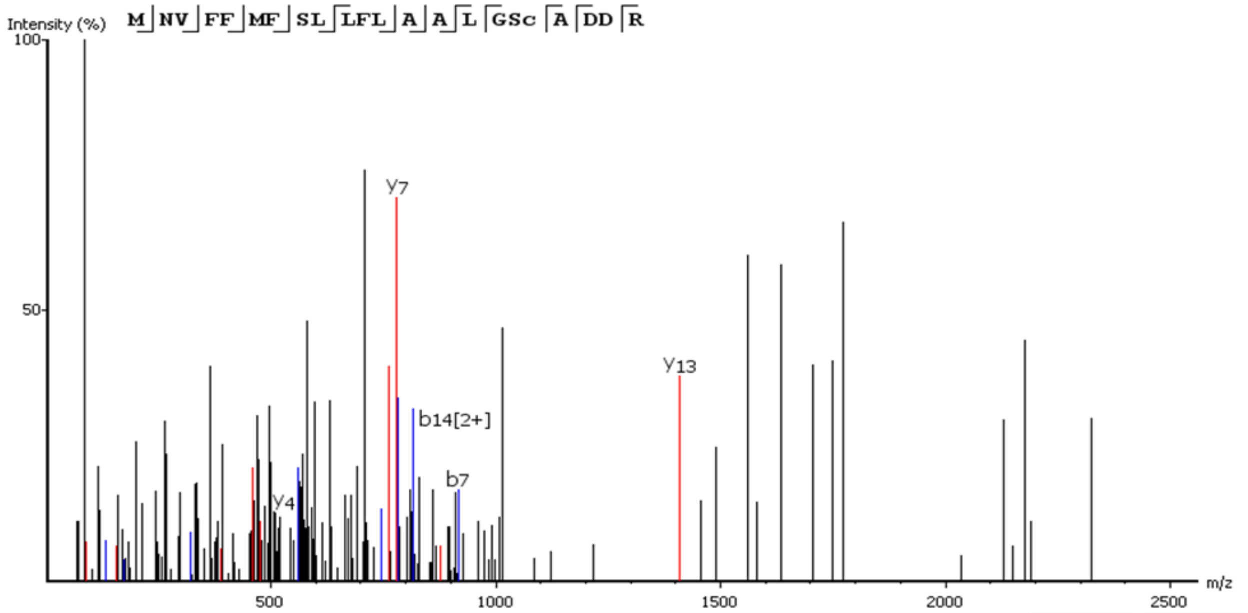

Figure S2. Partial amino acid sequence of *C. purpureomaculatus* LAAO identified from LC-MS/MS (Top panel). Highlighted amino acid (red) represents mutation in the peptide sequence compared to similar proteins from other snake species. Mass to charge ratio (M/Z) of high confidence peptide match obtained from LC-MS/MS for *C. purpureomaculatus* LAAO (bottom panel).
